# Testing for the association of the *KIAA1109/Tenr/IL2/IL21 *gene region with rheumatoid arthritis in a European family-based study

**DOI:** 10.1186/ar2654

**Published:** 2009-03-20

**Authors:** Vitor Hugo Teixeira, Celine Pierlot, Paola Migliorini, Alejandro Balsa, René Westhovens, Pilar Barrera, Helena Alves, Carlos Vaz, Manuela Fernandes, Dora Pascual-Salcedo, Stefano Bombardieri, Jan Dequeker, Timothy R Radstake, Piet Van Riel, Leo van de Putte, Antonio Lopes-Vaz, Thomas Bardin, Bernard Prum, François Cornélis, Elisabeth Petit-Teixeira, the European Consortium on Rheumatoid Arthritis Families

**Affiliations:** 1 GenHotel-EA3886, Evry University – Paris 7 University Medical School, AutoCure European Consortium member, 2 rue Gaston Crémieux, 91057 Evry-Genopole, France; 2 Faculty of Medicine, University of Coimbra, Rua Larga, 3004-504 Coimbra, Portugal; 3 Pisa University, Lungarno Pacinotti 43, 56126 Pisa, Italy; 4 La Paz Hospital, Paseo Castellana 261, 28046 Madrid, Spain; 5 KUL Leuven University, Naamsestraat 63, BE-3000 Leuven, Belgium; 6 Nijmegen University, Geert Grooteplein 10, 6500HB Nijmegen, The Netherlands; 7 Porto San Joao Hospital, Estrada Interior da Circunvalação, 4200 Porto, Portugal; 8 Rheumatology Federation in Pôle Appareil Locomoteur, Lariboisière Hospital, Assistance Publique-Hôpitaux de Paris, 2 rue Ambroise Paré, 75010 Paris, France; 9 Laboratoire Statistique et Génome, Genopole, Tour Evry 2, 91000 Evry, France; 10 Clinical Genetics Unit in Pôle Laboratoire–Imagerie–Pharmacie Lariboisière Hospital, Assistance Publique-Hôpitaux de Paris, 2 rue Ambroise Paré, 75010 Paris, France; 11 Adult Genetics Unit, Centre Hospitalier Sud Francilien, Evry-Corbeil, 59 bd H Dunant, 91106 Corbeil-Essonnes, France

## Abstract

**Introduction:**

A candidate gene approach, in a large case–control association study in the Dutch population, has shown that a 480 kb block on chromosome 4q27 encompassing *KIAA1109/Tenr/IL2/IL21 *genes is associated with rheumatoid arthritis. Compared with case–control association studies, family-based studies have the added advantage of controlling potential differences in population structure. Therefore, our aim was to test this association in populations of European origin by using a family-based approach.

**Methods:**

A total of 1,302 West European white individuals from 434 trio families were genotyped for the rs4505848, rs11732095, rs6822844, rs4492018 and rs1398553 polymorphisms using the TaqMan Allelic discrimination assay (Applied Biosystems). The genetic association analyses for each SNP and haplotype were performed using the Transmission Disequilibrium Test and the genotype relative risk.

**Results:**

We observed evidence for association of the heterozygous rs4505848-AG genotype with rheumatoid arthritis (*P *= 0.04); however, no significance was found after Bonferroni correction. In concordance with previous findings in the Dutch population, we observed a trend of undertransmission for the rs6822844-T allele and rs6822844-GT genotype to rheumatoid arthritis patients. We further investigated the five SNP haplotypes of the *KIAA1109/Tenr/IL2/IL21 *gene region. We observed, as described in the Dutch population, a nonsignificant undertransmission of the AATGG haplotype to rheumatoid arthritis patients.

**Conclusions:**

Using a family-based study, we have provided a trend for the association of the *KIAA1109/Tenr/IL2/IL21 *gene region with rheumatoid arthritis in populations of European descent. Nevertheless, we failed to replicate a significant association of this region in our rheumatoid arthritis family sample. Further investigation of this region, including detection and testing of all variants, is required to confirm rheumatoid arthritis association.

## Introduction

Rheumatoid arthritis (RA) is a common autoimmune inflammatory disease of unknown cause, in which both genetic and environmental risk factors have been implicated [1]. Since the genetic contribution to RA has been estimated to be between 50% and 60%, identification of genes contributing to disease is important for the understanding of underlying biological mechanisms [2].

In addition to human leucocyte antigen (*HLA*) [3] – the first identified genetic risk factor – and four other replicated regions – including the protein tyrosine phosphatase N22 gene (*PTPN22*) [4], the TNF receptor-associated factor 1 gene (*TRAF1*)/complement component factor 5 (*C5*) locus [5-7], the 6q23 locus near the TNFα-induced protein 3 gene (*TNFAIP3*) [8,9], and the signal transducer and activator of transcription 4 (*STAT4*) gene [10,11] – a new genetic region associated with RA was described in the Dutch population [12]. This region encompassing *KIAA1109/Tenr/IL2/IL21 *is contained in a large block (480 kb) of linkage disequilibrium located on chromosome 4q27 and includes the *IL2 *and *IL21 *genes, which are both plausible functional candidate loci for RA. Five SNPs (rs4505848, rs11732095, rs6822844, rs4492018 and rs1398553) of the block were investigated in a sample set consisting of 1,047 RA patients and 929 controls, showing a significant association of the rs6822844-T allele and the rs6822844-GT genotype (*P *= 0.0002, odds ratio (OR) = 0.72, 95% confidence interval (CI) = 0.61 to 0.86; and *P *= 0.0009, OR = 0.71, 95% CI = 0.58 to 0.87, respectively) and of a specific haplotype (AATGG) (*P *= 0.00025, OR = 0.70, 95% CI = 0.57 to 0.85) with RA [12].

Furthermore, in the North American Rheumatoid Arthritis Consortium and in the Epidemiological Investigation of Rheumatoid Arthritis populations, rs6822844 and rs1398553 SNPs were significantly associated with RA (*P *= 0.01, OR = 0.84, 95% CI = 0.74 to 0.96; and *P *= 0.003, OR = 1.17, 95% CI = 1.05 to 1.30, respectively) [6]. In addition, the last meta-analysis performed with the North American Rheumatoid Arthritis Consortium, the Epidemiological Investigation of Rheumatoid Arthritis and the Wellcome Trust Case Control Consortium studies, with 3,393 RA patients and 12,462 controls, observed evidence of association of 4q27 with RA in an independent replication, suggesting that 4q27 is a true-positive association [13].

Given that association studies compare the frequencies in patients versus healthy individuals, unknown biases in control frequencies may lead to spurious associations. Family-based association studies therefore remain important to definitively establish association, especially when small effect sizes as well as variability in allele frequency in different populations are observed. With the aim to further substantiate the association at the *KIAA1109/Tenr/IL2/IL21 *gene region, we therefore took advantage of one of the largest reported European family resources dedicated to RA family-based studies.

## Materials and methods

### Study design and study population

DNA was available from 434 white trio families from Western Europe through the European Consortium on Rheumatoid Arthritis Families collection followed by the selection of individuals fulfilling the American College of Rheumatology 1987 criteria for RA [14], according to the rheumatologist in charge of the patient. Each family consisted of one RA patient and both parents, with the ethnicity as determined by grandparental origin.

Characteristics of the 434 RA European Caucasian families are reported in Table 1. The 434 families included 308 families from France, 51 families from Italy, 32 families from Spain, 20 families from Belgium, nine families from the Netherlands and 14 families from Portugal. Families with an additional affected sibling and with RA patients aged <18 years were excluded. All individuals provided written informed consent and the study was approved by the Ethics Committee of Hôpital Kremlin-Bicêtre (Paris, France).

**Table 1:** Characteristics of rheumatoid arthritis (RA) index cases

Characteristic	RA families (n = 434)
Females (*n *(%))	375 (86%)
Mean age at disease onset (years) (± standard deviation)	31.1 (9.7)
Mean disease duration (years) (± standard deviation)	9.8(8.5)
With bone erosions (*n *(%))	336 (77%)
Seropositive for rheumatoid factor (*n *(%))	323 (76%) (out of 425 RA patients)
Seropositive for anti-cycle citrullinated peptides antibodies (*n *(%))	214 (76%) (out of 282 RA patients)
Carrying at least one *HLA-DRB1 *shared epitope allele (*n *(%))^a^	152 (77%) (out of 197 RA patients)
Carrying at least one PTPN22-1858T allele (*n *(%))	115 (27%)
Carrying the *TRAF1/C5 *rs10818488-A variant (*n *(%))	277 (64%)

^a^DRB1*0101, DRB1*0102, DRB1*0401, DRB1*0404, DRB1*0405, DRB1*0408, DRB1*1001.

### Genotyping

All samples were genotyped using the TaqMan allelic discrimination assay according to the manufacturer's instructions (Applied Biosystems, Foster City, CA, USA). Centre d'Etude du Polymorphisme Humain DNA samples were co-genotyped with all our samples, with no inconsistencies detected. The genotyping success rate in the 434 trio families was 100% (parents and probands included).

### Statistical analysis

Using the previously reported frequencies for the rs6822844-T allele in Dutch RA patients (14.1%) and in controls (18.5%) [12], the power to detect an association was calculated using the method described by Garnier and colleagues [15]. The Hardy–Weinberg equilibrium was checked in the control group (constituting the nontransmitted parental chromosomes from the trio) prior to analysis. The association analysis relied on the Transmission Disequilibrium Test, which for a given allele compares its transmission from heterozygous parents to RA patients with the transmission expected from Mendel's law (that is, 50%) [16].

Secondly, we used the genotype relative risk, which compares the affected offspring's genotype with the control genotype derived from nontransmitted parental chromosomes [17]. ORs were calculated and 95% CIs were approximated using Woolf's method [18], with Haldane's correction [19]. Haplotypes were generated using GENEHUNTER software [20]. We then carried out multi-locus tests of association testing haplotypes of SNPs using the Transmission Disequilibrium Test and haplotype relative risk. *P *values were adjusted with Bonferroni correction [21] for each independent test performed.

## Results

### Power calculation

Using the previously reported frequencies for the rs6822844-T allele in Dutch RA patients (14.1%) and in controls (18.5%) [12], association analysis of 434 trio families provides an 80% power to reach statistical significance (*P *<0.05).

### Hardy–Weinberg equilibrium check

The five SNPs in the sample investigated were in Hardy–Weinberg equilibrium in the control group.

### Test for association in RA trio families

A total of 1,302 European individuals from 434 trio families (one RA case and both parents) were analyzed. Three hundred and eight families were of French origin and 126 were from other continental Western European countries. With the rs6822844 (G/T) SNP we observed deviation from Mendel's first law, with a nonsignificant undertransmission of 45.3% of the T allele to the patients in the 434 families (*P *= 0.24) (Table 2). No significant association with RA was detected for the four other SNPs (Table 2).

**Table 2:** Family-based association of the chromosome 4q27 gene region with rheumatoid arthritis: Transmission Disequilibrium Test

4q27 region SNP	Allele	Transmitted allele	Untransmitted allele	Transmission^a ^(%)	*P *value
rs4505848	A	185	189	49.5	0.84
rs11732095	A	96	78	55.2	0.17
rs6822844	T	97	114	45.3	0.24
rs4492018	G	183	174	51.3	0.63
rs1398553	G	182	182	50	1

^a^Percentage of transmission from heterozygous parents.

Since RA is a heterogeneous disease and distinct subsets of patients are characterized by the presence of autoantibodies such a rheumatoid factor and anti-citrullinated protein antibodies, we also performed a stratified analysis for the presence or absence of rheumatoid factor or anti-citrullinated protein antibodies; no associations were detected (data not shown). Furthermore, the same tests were performed in subgroups stratified for the presence of the *HLA-DRB1 *shared epitope, *PTPN22*-1858T and *TRAF1/C5 *rs10818488-A variant, and no associations were detected (data not shown). One of the advantages of family trio data is that they provide perfectly matched controls for each patient investigated. Each patient chromosome, transmitted by a given parent, is perfectly matched for the population of origin with the untransmitted chromosome of each heterozygous parent. The genotype relative risk analysis of the rs4505848 (A/G) SNP showed an association of the heterozygous AG genotype with RA (*P *= 0.04, OR = 1.32, 95% CI = 1.01 to 1.72) (Table 3). The significance threshold was not reached, however, after Bonferroni correction. In the four other SNPs, no association with RA was detected (Table 3). In concordance with previous findings in the Dutch population, we observed an undertransmission of the rs6822844-T allele and the rs6822844-GT genotype, but without significance (Tables 2 and 3).

**Table 3:** Family-based association of the chromosome 4q27 gene region with rheumatoid arthritis: genotype relative risk

SNP	Genotype	RA cases (*n*)	Controls (*n*)	Odds ratio (95% confidence interval)	*P *value
rs4505848	GG	38	51	0.72 (0.46 to 1.12)	0.18 (GG vs. AA + AG)
	AG	213	183	1.32 (1.01 to 1.72)	0.04 (AG vs. GG + AA)^a^
	AA	182	199	0.85 (0.65 to 1.11)	0.28 (AA vs. AG + GG)
rs11732095	GG	7	9	0.77 (0.28 to 2.09)	0.8 (GG vs. AA + AG)
	AG	87	101	0.83 (0.6 to 1.15)	0.28 (AG vs. GG + AA)
	AA	340	324	1.23 (0.9 to 1.68)	0.23 (AA vs. AG + GG)
rs6822844	TT	8	11	0.72 (0.29 to 1.81)	0.64 (TT vs. GG + TG)
	TG	99	110	0.87 (0.64 to 1.19)	0.43 (TG vs. TT + GG)
	GG	327	313	1.18 (0.87 to 1.6)	0.32 (GG vs. TT + TG)
rs4492018	GG	235	232	1.03 (0.79 to 1.35)	0.89 (GG vs. AA + AG)
	AG	174	171	1.03 (0.78 to 1.35)	0.89 (AG vs. GG + AA)
	AA	25	31	0.79 (0.46 to 1.36)	0.49 (AA vs. AG + GG)
rs1398553	GG	204	214	0.91 (0.7 to 1.19)	0.54 (GG vs. AA + AG)
	AG	195	175	1.2 (0.92 to 1.57)	0.20 (AG vs. GG + AA)
	AA	35	45	0.77 (0.49 to 1.21)	0.30 (AA vs. AG + GG)

^a^*P *value was not significant after Bonferroni correction.

We further investigated the five SNP (rs4505848, rs11732095, rs6822844, rs4492018 and rs1398553) haplotypes of the *KIAA1109/Tenr/IL2/IL21 *gene region. These seven haplotypes of the combined five SNPs of the block have a pooled frequency of 96%. We observed a nonsignificant undertransmission of the AATGG haplotype (*P *= 0.19) (Table 4), which was reported as associated with RA in the Dutch study [12]. No relative risk was detected for any haplotype genotype (data not shown). There were no differences between families of French origin and other continental Western Europe families, as well as no differences between paternal transmission and maternal transmission for all statistical analyses performed (data not shown).

**Table 4:** Family-based association for the seven haplotypes^a^: Transmission Disequilibrium Test

Haplotype	Transmitted	Untransmitted	Transmission^b ^(%)	*P *value
GAGGA	113	112	50	0.95
AAGAG	133	126	52	0.69
AATGG	76	93	45	0.19
AAGGG	110	102	52	0.58
AGGGG	67	67	50	1
GAGGG	81	64	56	0.16
AAGGA	53	43	55	0.31

^a^rs4505848 (G/A) – rs11732095 (G/A) – rs6822844 (T/G) – rs4492018 (G/A) – rs1398553 (G/A). ^b^Percentage of transmission from heterozygous parents.

## Discussion

A recent case–control study in the Dutch population has shown association of RA, celiac disease and type 1 diabetes with the 4q27 gene region. In that study the rs6822844-T allele was reported to be a perfect proxy for a haplotype that is highly associated with autoimmune diseases with frequencies of 0.13 in cases versus 0.19 in North European controls [12].

In addition, a study from the Wellcome Trust Case Control Consortium identified this 4q27 region in a search for risk factors for type 1 diabetes [22]. In a follow-up study, some support for this association with type 1 diabetes was provided [23]. The last meta-analysis of data from three genome-wide association studies of type 1 diabetes – testing 305,090 SNPs in 3,561 type 1 diabetes cases and 4,646 controls of European ancestry – obtained further support for 4q27 type 1 diabetes association (*P *= 1.9 × 10^-8^), indicating that this is a true type 1 diabetes locus [24]. The same region was studied in inflammatory bowel diseases such as Crohn's disease and ulcerative colitis. Four SNPs were genotyped in three cohorts concerning 4,782 cases (3,194 ulcerative colitis, 1,588 Crohn's disease) and 2,616 controls. All four SNPs were strongly associated with ulcerative colitis and moderately associated with Crohn's disease [25]. The 4q27 locus was also reported to be associated with celiac disease [26]. Recent evidence is also provided of a role for this region in psoriasis and psoriatic arthritis [27]. Finally, a genetic association with systemic lupus erythematosus and two SNPs located within the *IL-21 *gene was found in a case–control study (1,318 cases and 1,318 controls) [28]. All of these studies provide evidence that 4q27 seems to be a common locus for multiple forms of autoimmune diseases.

In our family-based study, compared with the Dutch study, we have detected a similar undertransmission of the rs6822844-T allele, the rs6822844-GT genotype and the AATGG haplotype, but without significance. Furthermore, we observed evidence of association between RA and the rs4505848-AG genotype, but no significance was found after Bonferroni correction. These results exclude the 4q27 locus from being a major RA genetic region, but a minor association should be not excluded and needs to be tested in a larger RA trio sample. Furthermore the already cited last RA meta-analysis, suggesting a 4q27 true-positive association, was performed in a large number of individuals. For the rs4572894 SNP tested, there was only a 2% variation between RA and non-RA minor allele frequency (27% versus 29%, respectively) with a *P *value of 1.1% [13]. The putative effect of the 4q27 locus in RA is therefore very small and difficult to replicate in our RA family sample. In addition, a two-stage genome-wide association study of RA in the Spanish population did not found 4q27 as a RA susceptibility gene region [29].

The long region of linkage disequilibrium at chromosome 4q27 contains several genes: testis nuclear RNA-binding protein, a gene encoding a protein of unknown function (KIAA1109), and genes encoding the IL-2 and IL-21 cytokines. Testis nuclear RNA-binding protein is expressed primarily in the testis and KIAA1109 transcripts are ubiquitous, hence their roles in autoimmunity are not particularly compelling. Both IL-2 and IL-21 belong to the type 1 cytokine family, share a large degree of homology, and possess pleotropic functions in immune cells [26]. These two genes are both plausible functional candidates as genetic modifiers of autoimmunity, and thus have particular interest to RA.

IL-2 exerts its effects on many cell types, the most prominent of which is the T lymphocyte. Accordingly, a major function of IL-2 is to promote proliferation and expansion of both antigen-specific clones of CD4^+ ^and CD8^+ ^T cells as well as to induce production of other cytokines. In CD4^+ ^T cells, IL-2 plays a nonredundant role in the development of CD4^+ ^CD25^+ ^regulatory T cells. Accumulating evidence supports CD4^+ ^CD25^high ^regulatory T cells playing an essential role in controlling and preventing autoimmunity [30]. In addition, the *IL2 *receptor (*CD25*) susceptibility locus has recently been reported to be associated with RA [22].

IL-21 is involved in both cell-mediated and humoral responses and has a pleiotropic effect on a variety of immune and nonimmune cells. In RA, the synovial fluid and tissue have enhanced inflammatory responses to IL-21 and elevated IL-21 receptor expression. In two animal models – collagen-induced arthritis and adjuvant-induced arthritis – treatment with an IL-21-blocking agent ameliorated disease and/or reversed established disease, and also lowered levels of IL-6 and IL-17 [31].

## Conclusions

In the present study, using a family-based approach, we have provided a trend for the association of the *KIAA1109/Tenr/IL2/IL21 *gene region with RA in European descent populations. Nevertheless, we failed to replicate a significant association of this region in our RA family sample. Further investigation of this region, including detection and testing of all variants, is required to confirm RA association.

## Abbreviations

CI: confidence interval; IL: interleukin; OR: odds ratio; RA: rheumatoid arthritis; SNP: single nucleotide polymorphism.

## Competing interests

The authors declare that they have no competing interests.

## Authors' contributions

VHT carried out the molecular genetic studies. VHT and EP-T carried out the design of the study and drafted the manuscript. VHT, EP-T, BP and CP performed analysis of the data. PM, AB, RW, PB, HA, CV, MF, DP-S, SB, JD, TRR, PVR, LvdP, AL-V, TB and FC contributed to the recruitment of families and to the acquisition of clinical data. All authors read and approved the final manuscript.

## Authors' information

The European Consortium on Rheumatoid Arthritis Families initiated with funding from the European Commission (BIOMED2) is coordinated by FC.
